# Post-traumatic intraosseous leptomeningeal cyst

**DOI:** 10.1590/0100-3984.2016.0166

**Published:** 2018

**Authors:** Francisco Barbosa de Araújo Neto, Vinícius Martins Valois, Marco Vinícius Dias, Sérgio Furlan, Dalton Yukio Araújo Fugita

**Affiliations:** 1 Hospital Heliópolis - Radiologia, São Paulo, SP, Brazil

**Figure 1:**
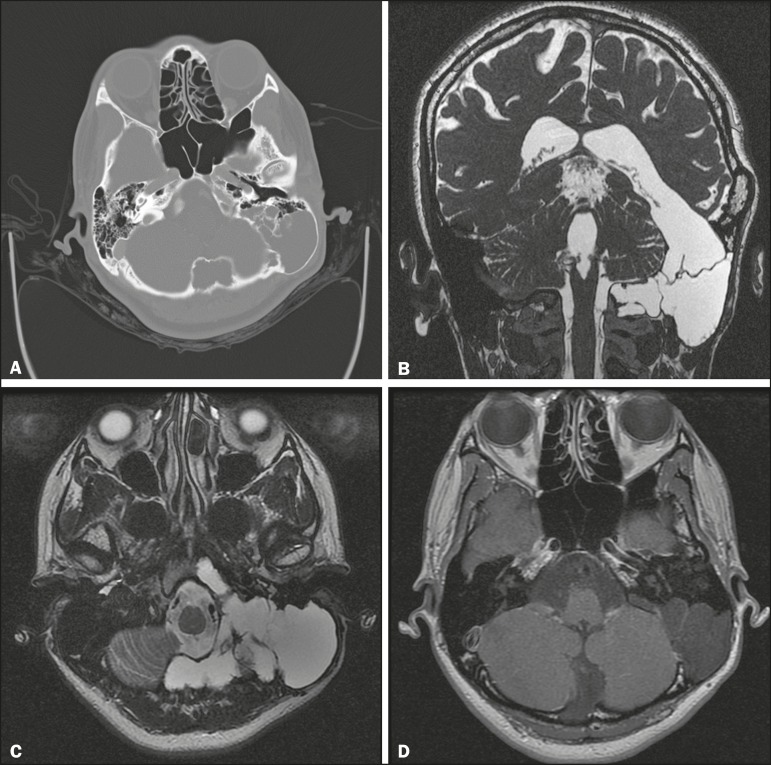
** A:** Axial CT, with bone window settings, showing cystic and expansile remodeling of the most posterior portions of the mastoid of the left temporal bone, with narrowing of the outer table of the skull, without rupture. **B:** Coronal MRI fast imaging employing steady-state acquisition sequence, showing leptomeningeal cystic communication extending from the left lateral ventricle, through the middle cranial fossa, toward the left temporal bone. **C:** T2-weighted turbo spin-echo MRI sequence, showing a leptomeningeal cyst, with bulging and expansion of the left temporal bone, as well as slight insinuation into the clivus. **D:** Contrast-enhanced T1-weighted MRI sequence showing a leptomeningeal cyst, with no anomalous enhancement or associated solid expansile lesions.

Dear Editor,

A 22-year-old female patient sought treatment at our facility with a three-year history of progressive left retroauricular bulging accompanied by mild pain, with no need for analgesics, and no other complaints. She also reported having suffered a head injury from a motor vehicle accident at six months of age. Computed tomography (CT) and magnetic resonance imaging (MRI) scans of the cranium revealed an intraosseous leptomeningeal cyst (Figure 1).

Post-traumatic intradiploic leptomeningeal cysts are extremely rare complications of calvarial fractures that occur during childhood^(1)^. The first case was reported by Weinand et al. in 1989^(2)^. They are also known by other names, including intraosseous leptomeningeal cysts^(3)^ and post-traumatic intradiploic pseudomeningoceles^(4)^. These cysts are characterized by fracture of the inner table of the skull and laceration of the dura mater, with accumulation of cerebrospinal fluid in a pouch lined with arachnoid membrane and located within the diploic space. The most common site is the occipital region, although such cysts have also been reported to occur in other regions of the skull^(5)^. The clinical presentation is highly variable, ranging from asymptomatic to calvarial defects to overlapping neurological complaints.

The most widely accepted hypothesis regarding the physiopathology of post-traumatic intradiploic leptomeningeal cysts is herniation of the leptomeninges to the diploic space through post-traumatic gaps in the dura mater and the inner table of the skull. After a traumatic incident, it can take weeks, months, or years for an intradiploic leptomeningeal cyst to develop^(6)^. Pressure effects and valve effects due to the growth of the brain during childhood, together with continuous cerebrospinal fluid pulses, act as expansive forces that promote the formation and growth of the intradiploic cyst over the years, resulting in wear and remodeling of the outer table of the skull^(7,8)^.

The radiological tools for the diagnosis of the leptomeningeal cyst are those that are also useful for the evaluation of cranial defects and associated brain lesions. An X-ray of the skull shows expansion of the diploic space and preservation of the outer table. A CT scan allows the evaluation of the extent of the bone defect and of the outer table of the skull, thus facilitating the surgical planning. However, MRI is the imaging modality of choice and is a valuable tool for identifying other lesions, such as dermoid and epidermoid cysts^(4)^.

The differential diagnoses include bone lesions such as myeloma, metastasis, and eosinophilic granuloma, as well as intradiploic arachnoid cyst, which is usually congenital and manifests as headache, edema, convulsions, or neurological deficit. Although it is difficult to distinguish between intradiploic arachnoid cyst and post-traumatic intradiploic leptomeningeal cyst via imaging methods, a history of trauma and the cyst being located in the occipital region favors a diagnosis of the latter.

Surgical intervention is the basis of the treatment for leptomeningeal cyst, and the indications for surgery include severe craniofacial deformity and persistent headache^(4,7)^. The surgical procedure involves duraplasty followed by cranioplasty with a calvarial bone graft.

In conclusion, we can infer that post-traumatic intradiploic leptomeningeal cyst is a rare condition, with variable neurological symptoms, occurring secondary to calvarial trauma occurring during childhood. Knowledge and early diagnosis of such cysts are important, because surgical intervention, when appropriate, can avoid neurological sequelae.
